# Trends and risk factors for infant mortality in the Lao People’s Democratic Republic

**DOI:** 10.1038/s41598-020-78819-9

**Published:** 2020-12-10

**Authors:** Viengsakhone Louangpradith, Eiko Yamamoto, Souphalak Inthaphatha, Bounfeng Phoummalaysith, Tetsuyoshi Kariya, Yu Mon Saw, Nobuyuki Hamajima

**Affiliations:** 1 Department of Healthcare Administration, Nagoya University Graduate School of Medicine, Nagoya, Japan; 2 Department of Healthcare and Rehabilitation, Ministry of Health, Vientiane, Lao People’s Democratic Republic; 3 Ministry of Health, Vientiane, Lao People’s Democratic Republic

**Keywords:** Paediatrics, Public health

## Abstract

A high infant mortality rate (IMR) indicates a failure to meet people’s healthcare needs. The IMR in Lao People’s Democratic Republic has been decreasing but still remains high. This study aimed to identify the factors involved in the high IMR by analyzing data from 53,727 live births and 2189 women from the 2017 Lao Social Indicator Survey. The estimated IMR decreased from 191 per 1000 live births in 1978–1987 to 39 in 2017. The difference between the IMR and the neonatal mortality rate had declined since 1978 but did not change after 2009. Factors associated with the high IMR in all three models (forced-entry, forward-selection, and backward-selection) of multivariate logistic regression analyses were auxiliary nurses as birth attendants compared to doctors, male infants, and small birth size compared to average in all 2189 women; and 1–3 antenatal care visits compared to four visits, auxiliary nurses as birth attendants compared to doctors, male infants, postnatal baby checks, and being pregnant at the interview in 1950 women whose infants’ birth size was average or large. Maternal and child healthcare and family planning should be strengthened including upgrading auxiliary nurses to mid-level nurses and improving antenatal care quality.

## Introduction

Infant mortality is a major health problem around the world. Globally, the estimated infant mortality in 2018 was 4.0 million, accounting for 75.5% of all deaths of children under 5-years^1^. While environmental factors play a role in infant mortality, biological problems such as preterm birth and congenital abnormalities are the most well-known causes of neonatal mortality^2,3^. Therefore, the infant mortality rate (IMR) can serve as a health indicator of whole populations and of countries’ socio-economic conditions^4^. Furthermore, the IMR can be used to analyze the availability, utilization, and efficacy of healthcare, especially for women during the prenatal period and parturition as well as for neonates and infants. A high IMR indicates a failure to meet people’s healthcare needs and suggests the presence of environmental factors unfavorable for infants. Predictive factors for infant mortality fall into three main categories: distal (socio-economic and demographic) factors; intermediate (maternal, reproductive, and healthcare delivery) factors; and proximal (neonatal) factors^5^. There are many reported predictive factors, and the results differ depending on the factors and locations^3,6,7^.

Lao People’s Democratic Republic (PDR) is a lower-middle income country in Southeast Asia. In 2018, the estimated population was 7,013,000, and approximately 70% of the population lived in rural areas^8,9^. To achieve the 2015 Millennium Development Goals, the Integrated Package of Maternal, Newborn and Child Health was implemented and incorporated in the free Maternal and Child Health (MCH) Policy, Skilled Birth Attendant Development Plan, Health Sector Reform Framework, and Health Sector Development Plan^10^. As a result of expanding health service delivery and improving healthcare quality, the IMR of Lao PDR declined; however, the improvement was slow^8,11^, and it remains the highest in Southeast Asia, except for Timor-Leste^12^. To achieve the Sustainable Development Goals^13^, continued efforts are needed in certain areas such as supervising skilled health workers; further increasing essential reproductive, maternal, and child health service coverage; providing adequate resources to facilities to implement the free MCH Policy; and empowering communities to take advantage of the available MCH services. The most recent national health survey, the Lao Social Indicator Survey II (LSIS-II), was conducted across a representative sample of the country’s population in 2017^8^. This study aimed to show the infant mortality trends and to identify factors associated with infant mortality by secondary analysis of the LSIS-II data.

## Results

### Estimation of the IMR and neonatal mortality rate (NMR)

**Figure 1:**
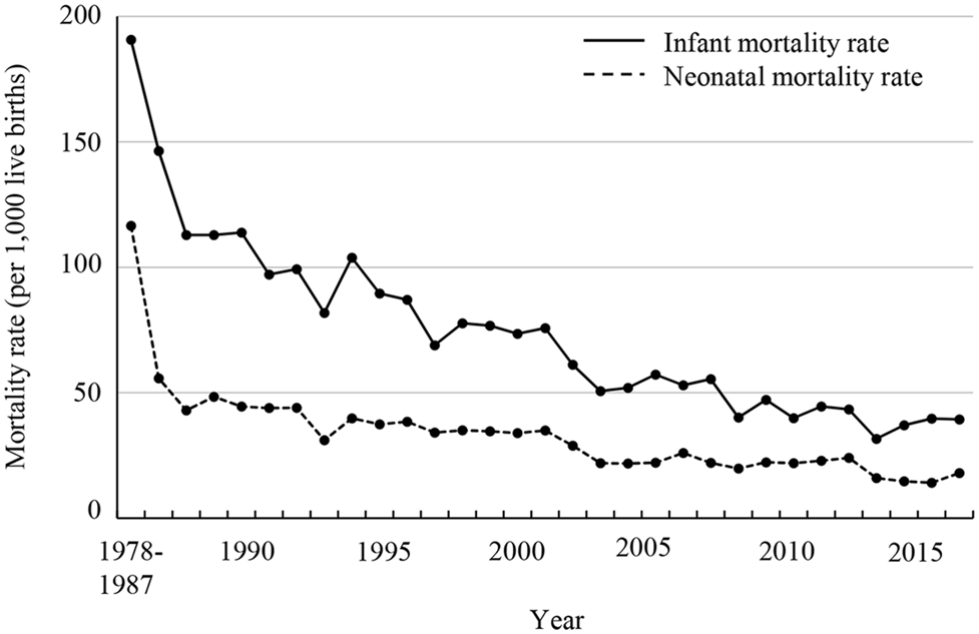
The trends of infant mortality rate (IMR) and neonatal mortality rate (NMR) from 1978 to 2017 in Lao PDR. The estimated IMR and NMR gradually decreased from 1978 to 2017. The difference between the IMR and NMR had been declining since 1978, however, this decreasing trend stopped after 2009.

To estimate the IMR and NMR in Lao PDR, we analyzed data from 53,727 children of women who participated in the LSIS-II. The children were born between 1978 and 2017. The IMR (per 1000 live births) was 191 in 1978–1987, decreasing to 39 in 2017 (Fig. 1). The NMR (per 1000 live births) rapidly decreased from 117 in 1978–1987 to 43 in 1989 and then slowly declined to 18 in 2017. Neonatal mortality accounted for 61.1% of the infant mortality in 1978–1987 and 38–56% in 1988–2017. The difference between the IMR and NMR decreased from 7.4 per 1000 live births in 1978–1987 to 2.0 in 2009; however, this decreasing trend stopped after 2009.

### Mortality of children born in the 2 years before LSIS-II

**Figure 2:**
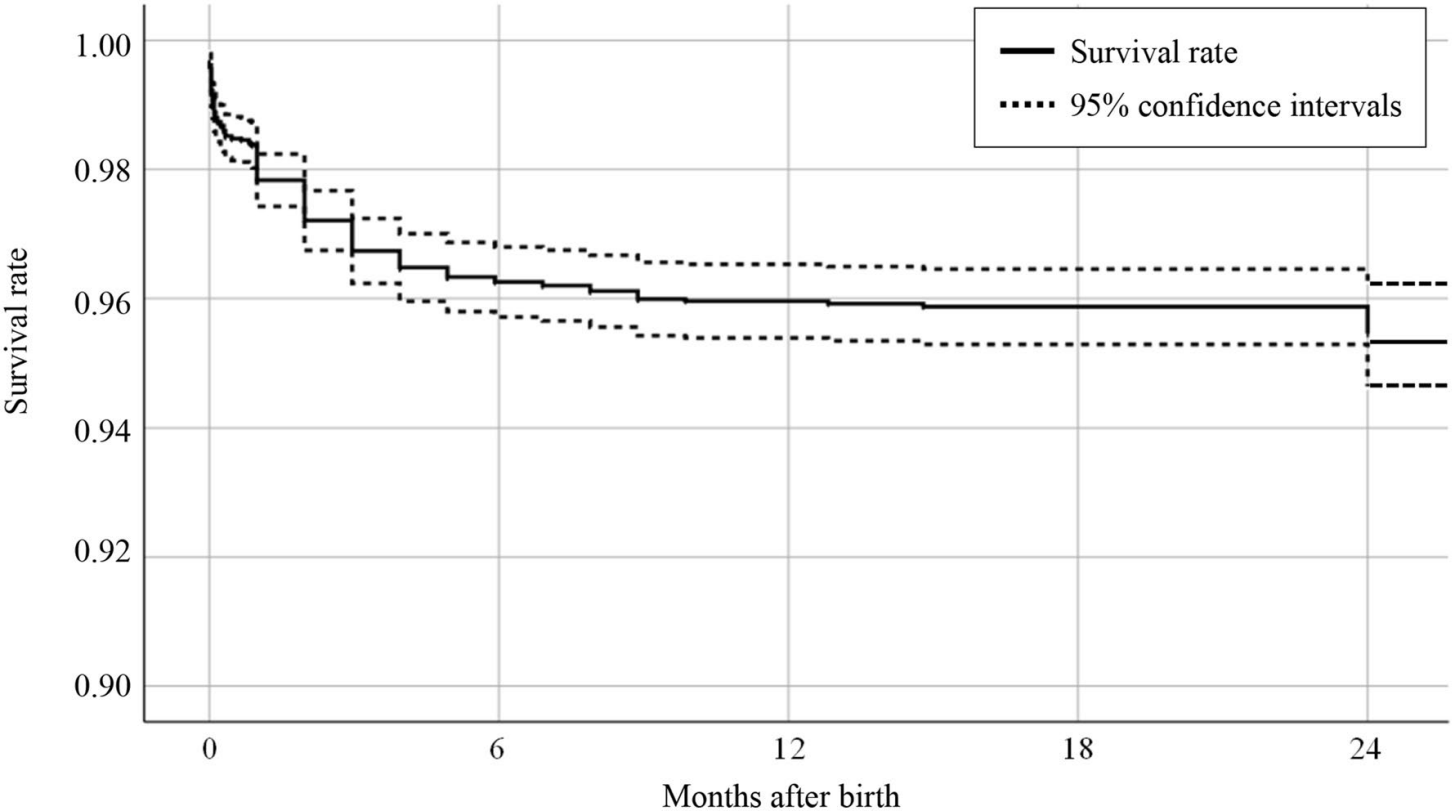
The survival rates of 5013 children who were born 2 years before the interview. The survival rate was 97.8% at 1 month, 96.0% at 1 year, and 95.5% at 2 years of life.

Next, we analyzed the survival time of children born in the 2 years before LSIS-II, using data from 5013 children (unweighted cases) whose birth dates, ages at the time of the interviews, and death ages were available. The overall survival rate of the 5013 children was 95.5% in the first 2 years of life (Fig. 2). The survival rate was 97.8% at 1 month of age and gradually decreased to 96.0% at 1 year. The decrease in the survival rate was the greatest in the first month of life, especially in the first 3 days.

### Factors associated with high infant mortality among all women

We focused on infant mortality as it is exogenous mortality, which is influenced by environmental factors such as socio-demographics and health service utilization. Of the 4460 women (unweighted cases) who had live births in the 2 years before the interviews, 2189 women were included in the analysis of factors associated with infant mortality. The last child of each of these women was a singleton and 1 year or older if alive or had died before the interviews. There were 148 children who had died before 1 year, including 17 (11.4%) and 62 children (41.6%) who died within 24 h and 28 days after birth, respectively. After using sampling weights, the IMR in this dataset was 68 per 1000 live births.

A binary logistic regression analysis was performed to compare the characteristics of the 2189 women (unweighted cases). Concerning socio-demographic characteristics, women who were younger than 18 years, resided in rural areas, were from the Mon-Khmer ethnic group, had low education levels, were from poor households, had husbands younger than 18 years, and were younger than 18 years at their first marriage reported significantly higher infant mortalities than did their counterparts (Table 1). There were nine obstetrical factors that were associated with high infant mortality: parity of six or more, being pregnant at the time of the interview, hemoglobin (Hb) < 11.0 g/dL, fewer than four antenatal care (ANC) visits, ANC providers other than doctors, no tetanus immunization during the last pregnancy, delivery location other than health facilities, birth attendant other than doctors, and vaginal delivery (Table 2). Concerning the neonatal factors, male babies and small-sized babies at birth (by mothers’ perceptions) had 1.48- and 3.77-times higher infant mortalities than did female and average-sized babies, respectively (Table 3). Women who approved of husbands’ violence toward their wives showed higher infant mortality, but the only women who showed significant differences in infant mortality were those who approved of violence toward a wife who refused sex with her husband and who burned food (Table 3).

**Table 1:** Comparison of socio-demographic factors according to infant mortality.

Variables	All women			Women with average or large babies at birth		
	IM^a^ (%)	OR (95% CI)	AOR^b^ (95% CI)	IM^a^ (%)	OR (95% CI)	AOR^c^ (95% CI)
**Age (years old)**						
15–17	17.0	1 (reference)	1 (reference)	12.8	1 (reference)	1 (reference)
18–29	6.2	0.31 (0.15–0.66)**	0.55 (0.21–1.41)	3.2	0.34 (0.13–0.88)*	0.59 (0.17–1.99)
30–39	7.1	0.36 (0.17–0.77)**	0.64 (0.21–1.96)	6.3	0.44 (0.16–1.17)	0.94 (0.23–3.87)
40–49	6.8	0.32 (0.10–1.04)	0.40 (0.08–2.00)	4.6	0.28 (0.06–1.29)	0.43 (0.06–3.34)
**Region**						
North	6.4	1 (reference)	1 (reference)	5.0	1 (reference)	1 (reference)
Centre	6.9	1.10 (0.74–1.63)	1.20 (0.74–1.95)	5.7	1.15 (0.72–1.81)	1.46 (0.81–2.63)
South	6.9	1.10 (0.68–1.80)	0.97 (0.54–1.74)	5.4	1.10 (0.61–1.97)	1.16 (0.57–2.35)
**Residence**^**d**^						
Urban	4.0	1 (reference)	1 (reference)	2.7	1 (reference)	1 (reference)
Rural with roads	7.6	1.97 (1.25–3.12)**	1.42 (0.80–2.53)	6.3	2.42 (1.37–4.27)**	1.80 (0.88–3.68)
Rural without roads	9.2	2.44 (1.35–4.44)**	1.66 (0.77–3.57)	8.6	3.30 (1.63–6.69)**	2.51 (1.00–6.30)
**Ethno-linguistic group**^**e**^						
Lao-Tai	5.7	1 (reference)	1 (reference)	4.3	1 (reference)	1 (reference)
Mon-Khmer	9.1	1.67 (1.14–2.44)**	1.07 (0.64–1.80)	8.0	1.98 (1.27–3.09)**	1.55 (0.85–2.82)
Hmong-Mien	6.9	1.26 (0.75–2.10)	0.92 (0.46–1.85)	6.2	1.46 (0.81–2.64)	1.37 (0.62–3.06)
Chinese-Tibetan	5.0	0.85 (0.25–2.82)	0.55 (0.14–2.22)	5.5	1.26 (0.37–4.25)	0.86 (0.20–3.76)
Others	9.5	1.37 (0.27–7.01)	1.06 (0.17–6.44)	0.0	–	–
**Education**						
None/ECE	9.7	1 (reference)	1 (reference)	7.7	1 (reference)	1 (reference)
Primary	7.9	0.80 (0.52–1.23)	0.88 (0.53–1.46)	6.7	0.85 (0.51–1.40)	0.90 (0.50–1.62)
Secondary	5.2	0.52 (0.32–0.84)**	0.57 (0.30–1.08)	4.0	0.50 (0.28–0.88)*	0.59 (0.28–1.23)
Higher	2.9	0.27 (0.12–0.63)**	0.47 (0.16–1.35)	2.2	0.27 (0.10–0.70)**	0.54 (0.16–1.89)
**Wealth index**^**f**^						
Poorest	9.8	1 (reference)	1 (reference)	8.2	1 (reference)	1 (reference)
Poorer	8.1	0.81 (0.52–1.27)	0.95 (0.56–1.59)	7.9	0.96 (0.58–1.59)	1.15 (0.64–2.07)
Middle	4.9	0.47 (0.27–0.82)**	0.76 (0.38–1.49)	4.0	0.46 (0.25–0.87)*	0.67 (0.31–1.46)
Richer	5.0	0.48 (0.28–0.84)*	1.18 (0.55–2.54)	3.2	0.36 (0.18–0.72)**	0.87 (0.34–2.26)
Richest	4.7	0.45 (0.26–0.80)*	1.58 (0.62–4.04)	3.2	0.37 (0.19–0.74)**	1.26 (0.40–4.01)
**Marital status**						
Married	6.8	1 (reference)	1 (reference)	5.4	1 (reference)	1 (reference)
Single/divorced	5.7	0.79 (0.24–2.62)	0.42 (0.06–2.84)	6.3	1.11 (0.33–3.75)	0.81 (0.10–6.71)
**Husband age (years old)**						
15–17	21.1	1 (reference)	1 (reference)	17.6	1 (reference)	1 (reference)
18–29	7.3	0.32 (0.10–1.04)	0.55 (0.13–2.36)	5.8	0.33 (0.08–1.29)	0.78 (0.14–4.21)
30–39	6.1	0.26 (0.08–0.86)*	0.44 (0.10–1.97)	5.2	0.24 (0.05–1.05)	0.64 (0.11–3.65)
40–49	6.6	0.29 (0.08–1.01)	0.37 (0.07–1.89)	4.3	0.35 (0.06–2.07)	0.36 (0.05–2.46)
**Age at first marriage (years old)**						
15–17	7.9	1 (reference)	1 (reference)	6.8	1 (reference)	1 (reference)
18–29	5.9	0.71 (0.50–0.99)*	0.99 (0.65–1.49)	4.3	0.61 (0.40–0.92)*	0.77 (0.47–1.24)
30–39	12.1	1.47 (0.49–4.47)	3.17 (0.78–12.91)	13.3	1.92 (0.62–5.91)	2.93 (0.61–14.02)

IM infant mortality, OR odds ratio, AOR adjusted odds ratio, CI confidence interval, ECE, early childhood education.

*P < 0.05. **P < 0.01.

^a^Sampling weights were applied.

^b^Adjusted for all variables included in Tables 1, 2, 3.

^c^Adjusted for all variables included in Tables 1, 2, 3 except cesarean delivery.

^d^A village is classified as urban, rural with roads, or rural without roads based on its characteristic features^42^.

^e^The ethno-linguistic groups were based on the ethnic categorization by the Lao government^43^.

^f^The wealth index is a composite indicator of wealth^8^.

**Table 2:** Comparison of obstetrical factors according to infant mortality.

Variables	All women			Women with average or large babies at birth		
	IM^a^ (%)	OR (95% CI)	AOR^b^ (95% CI)	IM^a^ (%)	OR (95% CI)	AOR^c^ (95% CI)
**Parity**						
1	7.9	1 (reference)	1 (reference)	5.9	1 (reference)	1 (reference)
2	5.5	0.68 (0.44–1.05)	0.73 (0.43–1.24)	4.2	0.69 (0.41–1.17)	0.56 (0.30–1.06)
3–5	6.0	0.75 (0.49–1.17)	0.69 (0.37–1.28)	5.4	0.89 (0.53–1.49)	0.65 (0.31–1.36)
6 or more	12.0	1.59 (0.89–2.86)*	1.37 (0.55–3.42)	10.7	1.92 (0.98–3.78)	1.21 (0.41–3.55)
**History of miscarriage**						
No	6.9	1 (reference)	1 (reference)	5.4	1 (reference)	1 (reference)
Yes	6.3	0.90 (0.52–1.57)	0.79 (0.43–1.47)	5.7	1.04 (0.56–1.94)	0.95 (0.47–1.88)
**History of stillbirth**						
No	6.7	1 (reference)	1 (reference)	5.5	1 (reference)	1 (reference)
Yes	8.2	1.26 (0.50–3.18)	2.03 (0.71–5.75)	3.7	0.80 (0.22–2.95)	1.21 (0.30–4.94)
**History of abortion**						
No	6.7	1 (reference)	1 (reference)	5.3	1 (reference)	1 (reference)
Yes	7.7	1.10 (0.53–2.25)	1.31 (0.57–3.00)	7.5	1.45 (0.69–3.06)	2.28 (0.96–5.44)
**Pregnant at the time of the interview**						
No/don’t know	6.3	1 (reference)	1 (reference)	5.1	1 (reference)	1 (reference)
Yes	12.1	2.02 (1.21–3.38)**	1.53 (0.84–2.79)	10.0	2.10 (1.14–3.85)*	2.16 (1.08–4.32)*
**History of smoking**						
No	6.7	1 (reference)	1 (reference)	5.4	1 (reference)	1 (reference)
Yes	7.6	1.14 (0.61–2.10)	0.91 (0.45–1.81)	6.8	1.22 (0.59–2.51)	0.88 (0.39–2.00)
**History of drinking**						
No	6.6	1 (reference)	1 (reference)	4.8	1 (reference)	1 (reference)
Yes	6.8	1.01 (0.65–1.58)	1.61 (0.93–2.79)	5.6	1.20 (0.69–2.08)	2.26 (1.16–4.39)*
**Hemoglobin level (g/dL)**						
≥ 11.0	5.1	1 (reference)	1 (reference)	4.0	1 (reference)	1 (reference)
< 11.0	11.0	2.28 (1.33–3.90)**	1.62 (0.89–2.96)	8.6	2.30 (1.21–4.37)*	1.93 (0.93–4.00)
**Wanted the last child**						
Yes	6.7	1 (reference)	1 (reference)	5.3	1 (reference)	1 (reference)
No	6.8	1.05 (0.61–1.81)	1.07 (0.59–1.96)	6.9	1.27 (0.69–2.34)	1.29 (0.65–2.57)
**Antenatal care visits**						
None	9.2	1.88 (1.24–2.84)**	1.12 (0.57–2.16)	7.4	1.97 (1.20–3.22)**	0.83 (0.38–1.84)
1–3 times	9.5	1.94 (1.27–2.97)**	1.59 (0.97–2.59)	9.1	2.41 (1.49–3.91)***	1.77 (1.01–3.08)*
≥ 4 times	5.1	1 (reference)	1 (reference)	4.0	1 (reference)	1 (reference)
**Antenatal care provider**						
Doctor	4.9	1 (reference)	1 (reference)	4.0	1 (reference)	1 (reference)
Nurse/midwife	8.7	1.85 (1.18–2.89)**	1.35 (0.76–2.38)	7.0	1.85 (1.10–3.12)*	1.21 (0.64–2.40)
Auxiliary nurse	13.9	2.96 (1.11–8.07)*	1.51 (0.45–5.12)	12.5	3.60 (1.22–10.62)*	1.38 (0.37–5.22)
Others	10.4	2.11 (0.95–4.67)**	1.29 (0.50–3.33)	7.6	2.13 (0.83–5.42)	1.13 (0.36–3.55)
**Iron supplementation during the last pregnancy**						
Yes	4.5	1 (reference)	1 (reference)	2.5	1 (reference)	1 (reference)
No/don’t know	7.2	1.64 (0.97–2.80)	1.24 (0.69–2.34)	6.0	2.47 (1.20–5.09)*	1.96(0.90–4.27)
**Tetanus toxoid vaccination during the last pregnancy**						
Yes	5.2	1 (reference)	1 (reference)	4.2	1 (reference)	1 (reference)
No/don’t know	8.3	1.64 (1.16–2.34)**	1.54 (1.01–2.34)*	6.7	1.64 (1.08–2.47)*	1.46 (0.89–2.38)
**Delivery location**						
Health facility	5.2	1 (reference)	1 (reference)	3.9	1 (reference)	1 (reference)
Home	9.1	1.83 (1.29–2.59)**	0.86 (0.31–2.39)	8.4	2.21 (1.48–3.31)***	0.83 (0.24–2.84)
Others	20.8	4.75 (1.71–13.15)**	3.01 (1.80–11.66)	5.3	1.23 (0.15–10.32)	0.87 (0.08–9.44)
**Birth attendant**						
Doctor	4.3	1 (reference)	1 (reference)	3.1	1 (reference)	1 (reference)
Nurse/midwife	8.2	1.99 (1.11–3.58)*	1.38 (0.66–2.88)	5.6	1.85 (0.89–3.81)	1.11 (0.45–2.74)
Auxiliary nurse	23.5	7.57 (2.49–23.01)***	6.91 (1.77–27.05)**	23.5	10.50 (3.39–32.46)***	10.02 (2.39–41.94)**
No/others	9.6	2.35 (1.62–3.43)***	1.74 (0.60–5.08)	8.6	2.90 (1.85–4.53)***	2.84 (0.68–9.00)
**Cesarean delivery**						
Yes	1.6	1 (reference)	1 (reference)	0.0	–	–
No/don’t know	7.1	4.01 (1.11–14.45)*	3.34 (0.83–13.52)	5.8	–	–

IM infant mortality, OR odds ratio, AOR adjusted odds ratio, CI confidence interval.

*P < 0.05. **P < 0.01. ***P < 0.001.

^a^Sampling weights were applied.

^b^Adjusted for all variables included in Tables 1, 2, 3.

^c^Adjusted for all variables included in Tables 1, 2, 3 except cesarean delivery.

**Table 3:** Comparison of neonatal factors and attitude towards domestic violence according to infant mortality.

Variables	All women			Women with average or large babies at birth		
	IM^a^ (%)	OR (95% CI)	AOR^b^ (95% CI)	IM^a^ (%)	OR (95% CI)	AOR^c^ (95% CI)
**Infant sex**						
Male	7.9	1.48 (1.04–2.11)*	1.64 (1.12–2.39)*	6.6	1.68 (1.10–2.56)*	1.94 (1.23–3.07)**
Female	5.4	1 (reference)	1 (reference)	4.1	1 (reference)	1 (reference)
**Infant size at birth**						
Average	5.5	1 (reference)	1 (reference)	5.5	1 (reference)	1 (reference)
Small	17.8	3.77 (2.45–5.82)***	3.16 (1.95–5.11)***	–	–	–
Larger	5.2	0.92 (0.52–1.63)	1.01 (0.55–1.85)	5.2	0.92 (0.52–1.63)	0.87 (0.46–1.63)
**Postnatal baby check**						
Yes	7.8	1 (reference)	1 (reference)	6.7	1 (reference)	1 (reference)
No/don’t know	6.6	0.83 (0.52–1.34)	0.64 (0.37–1.12)	5.2	0.75 (0.44–1.28)	0.47 (0.25–0.90)*
**Substance consumed by babies in the first 3 days after birth**						
Breast milk/formula	6.4	1 (reference)	1 (reference)	5.2	1 (reference)	1 (reference)
Others	7.6	1.19 (0.82–1.72)	1.38 (0.89–2.13)	6.1	1.15 (0.75–1.78)	1.45 (0.87–2.42)
**Beating wife is justified if she goes out without telling husband**						
No	6.4	1 (reference)	1 (reference)	4.9	1 (reference)	1 (reference)
Yes/don’t know	8.2	1.30 (0.87–1.95)	1.17 (0.63–2.18)	7.7	1.62 (1.03–2.54)*	1.48 (0.74–2.93)
**Beating wife is justified if she neglects children**						
No	6.4	1 (reference)	1 (reference)	5.0	1 (reference)	1 (reference)
Yes/don’t know	7.8	1.24 (0.85–1.82)	0.96 (0.50–1.83)	6.8	1.39 (0.90–2.16)	0.94 (0.45–1.96)
**Beating wife is justified if she argues with her husband**						
No	6.3	1 (reference)	1 (reference)	5.0	1 (reference)	1 (reference)
Yes/don’t know	8.3	1.34 (0.92–1.95)	1.08 (0.60–1.95)	7.0	1.45 (0.94–2.23)	0.89 (0.44–1.71)
**Beating wife is justified if she refuses sex with her husband**						
No	6.3	1 (reference)	1 (reference)	4.8	1 (reference)	1 (reference)
Yes/don’t know	9.3	1.52 (0.99–2.34)*	1.35 (0.72–2.53)	9.1	1.93 (1.20–3.09)**	1.82 (0.89–3.73)
**Beating wife is justified if she burns food**						
No	6.4	1 (reference)	1 (reference)	5.1	1 (reference)	1 (reference)
Yes/don’t know	10.6	1.72 (1.01–2.93)*	1.00 (0.46–2.15)	9.1	1.86 (1.02–3.42)*	0.99 (0.43–2.28)

IM infant mortality, OR odds ratio, AOR adjusted odds ratio, CI confidence interval.

*P < 0.05. **P < 0.01. ***P < 0.001.

^a^Sampling weights were applied.

^b^Adjusted for all variables included in Tables 1, 2, 3.

^c^Adjusted for all variables included in Tables 1, 2, 3 except cesarean section.

Multivariable logistic regression analyses using all variables showed that not having a tetanus immunization during the last pregnancy (adjusted odds ratio (AOR) = 1.54), auxiliary nurses as birth attendants compared to doctors (AOR = 6.91), male babies (AOR = 1.64), and babies being small at birth compared to average (AOR = 3.16) were significantly associated with higher infant mortality (Tables 1, 2, 3).

### Factors associated with infant mortality among women whose babies were average or large at birth

It is well known that infants with a low birth weight have higher mortality and morbidity^14,15^. In this study, infants’ birth size (small, average, and large) by their mothers’ perceptions was used instead of birth weight. Therefore, we analyzed data of 1950 women (unweighted cases) whose babies were average or large at birth to determine the factors other than small size at birth that were associated with infant mortality. A binary logistic regression analysis was performed on infant mortality. Concerning socio-demographic factors, aged 15–17, residing in rural areas, being a member of the Mon-Khmer ethno-linguistic group, having no or early childhood education, and being part of the poorest wealth index were associated with high infant mortality (Table 1). Women who were first married at 18–29 years had significantly lower infant mortality than those were married at 15–17 years. Concerning obstetrical and neonatal factors, women who were pregnant at the time of the interview, had Hb < 11.0 g/dL, fewer than four ANC visits, ANC providers other than doctors, no iron supplementation or tetanus immunization during pregnancy, home births, auxiliary nurses or others as birth attendants, male babies, and postnatal baby checks had infant mortalities that were significantly higher than those of other women (Tables 2 , 3). Women who had cesarean sections had no infant deaths. Regarding attitudes toward domestic violence, women who accepted husbands’ violence toward their wives when she goes out without informing him, refuses sex with him, and burns food were similar to the results of all women (Table 3).

Multivariate logistic regression analysis of all factors, except delivery mode, revealed six factors associated with high infant mortality. Women who were pregnant at the time of the interview (AOR = 2.16) and women who consumed alcohol (AOR = 2.26) had higher infant mortalities than did others (Tables 1, 2, 3). Concerning ANC visits, the infant mortality of women who had 1–3 visits was 1.77-times higher than that of women who had four or more visits. Compared to doctors as birth attendants, only care by auxiliary nurses was associated with significantly higher infant mortality (AOR = 10.02). Male babies (AOR = 1.94) had significantly higher infant mortality than female babies, and babies who had postnatal checks had significantly lower infant mortality (AOR = 0.47) than that of the others. Two of the six associated factors—auxiliary nurses as attendants and male babies—produced the same results in the analysis of all women.

Forward-selection stepwise (Model 2) and backward-selection stepwise (Model 3) regression were applied for variable selection, while the forced-entry method (Model 1) used all variables for adjustment. The results of analyses were compared in the three models. Factors associated with high infant mortality in all three models were (1) having auxiliary nurses as birth attendants compared to doctors, male infants, and small infants in all women (Table 4); and (2) being pregnant at the time of interview, 1–3 ANC visits compared to four or more, having auxiliary nurses as birth attendants compared to doctors, having male infants, and having a postnatal baby check in women whose babies were average or large at birth (Table 5).

**Table 4:** Results of multivariate logistic regression models among all women.

Variables	Model 1	Model 2	Model 3
	AOR (95% CI)	AOR (95% CI)	AOR (95% CI)
**Pregnant at the time of the interview**			
No/don’t know	1 (reference)	1 (reference)	–
Yes	1.53 (0.84–2.79)	1.77 (1.03–3.05)*	–
**Hemoglobin level (g/dL)**			
≥ 11.0	1 (reference)	–	1 (reference)
< 11.0	1.62 (0.89–2.96)	–	1.85 (1.06–3.24)*
**Tetanus toxoid vaccination during the last pregnancy**			
Yes	1 (reference)	–	1 (reference)
No/don’t know	1.54 (1.01–2.34)*	–	1.45 (1.00–2.10)
**Birth attendant**			
Doctor	1 (reference)	1 (reference)	1 (reference)
Nurse/midwife	1.38 (0.66–2.88)	1.95 (1.07–3.54)*	1.94 (1.06–3.54)*
Auxiliary nurse	6.91 (1.77–27.05)**	9.48 (2.96–30.36)***	7.49 (2.36–23.78)***
No/others	1.74 (0.60–5.08)	2.15 (1.45–3.17 ***	1.89 (1.25–2.85)**
**Infant sex**			
Male	1.64 (1.12–2.39)*	1.66 (1.15–2.38)**	1.64 (1.14–2.36)**
Female	1 (reference)	1 (reference)	1 (reference)
**Infant size at birth**			
Average	1 (reference)	1 (reference)	1 (reference)
Small	3.16 (1.95–5.11)***	3.57 (2.28–5.58)***	3.75 (2.39–5.90) ***
Larger	1.01 (0.55–1.85)	0.98 (0.55–1.74)	1.01 (0.57–1.81)

AOR adjusted odds ratio, CI confidence interval. Model 1: forced-entry, Model 2: forward-selection, Model 3: backward-selection.

*P < 0.05. **P < 0.01. ***P < 0.001.

**Table 5:** Results of multivariate logistic regression models among women with average or large babies at birth.

Variables	Model 1	Model 2	Model 3
	AOR (95% CI)	AOR (95% CI)	AOR (95% CI)
**Pregnant at the time of the interview**			
No/don’t know	1 (reference)	1 (reference)	1 (reference)
Yes	2.16 (1.08–4.32)*	2.38 (1.27–4.47)**	2.06 (1.08–3.94)*
**History of drinking**			
No	1 (reference)	–	–
Yes	2.26 (1.16–4.39)*	–	–
**Hemoglobin level (g/dL)**			
≥ 11.0	1 (reference)	–	1 (reference)
< 11.0	1.93 (0.93–4.00)	–	2.02 (1.02–3.97)*
**ANC visits**			
None	0.83 (0.38–1.84)	1.14 (0.63–2.04)	1.01 (0.56–1.84)
1–3 times	1.77 (1.01–3.08)*	2.05 (1.23–3.43)**	2.03 (1.21–3.40)**
≥ 4 times	1 (reference)	1 (reference)	1 (reference)
**Birth attendant**			
Doctor	1 (reference)	1 (reference)	1 (reference)
Nurse/midwife	1.11 (0.45–2.74)	1.78 (0.85–3.73)	1.66 (0.78–3.49)
Auxiliary nurse	10.02 (2.39–41.94)**	13.56 (4.26–43.19)***	12.88 (4.03–41.20)***
No/others	2.84 (0.68–9.00)	2.82 (1.68–4.75)***	2.72 (1.61–4.60)***
**Infant sex**			
Male	1.94 (1.23–3.07)**	1.75 (1.14–2.70)*	1.75 (1.14–2.69)*
Female	1 (reference)	1 (reference)	1 (reference)
**Postnatal baby check**			
Yes	1 (reference)	1 (reference)	1 (reference)
No/don’t know	0.47 (0.25–0.90)*	0.56 (0.32–0.98)*	0.52 (0.29–0.92)*
**Beating wife is justified if she refuses sex with her husband**			
No	1 (reference)	1 (reference)	1 (reference)
Yes/don’t know	1.82 (0.89–3.73)	1.80 (1.11–2.94)*	1.79 (1.10–2.93)*

ANC antenatal care, AOR adjusted odds ratio, CI confidence interval. Model 1: forced-entry, Model 2: forward-selection, Model 3: backward-selection.

*P < 0.05. **P < 0.01. ***P < 0.001.

## Discussion

The results of this study are representative of Lao women and children because of the sampling method of LSIS-II, and the sampling weight was set accordingly. The findings are valuable for Lao PDR, where birth and death are not registered with the government correctly. The estimated IMR and NMR gradually decreased from 1978 to 2017. However, the IMR still remains high and a decrease of the difference between the IMR and NMR stopped after 2009. This may be attributable to a thiamine deficiency, which is associated with sudden infant death syndrome^16^ and may contribute to high infant mortality^17–20^. Severe thiamine deficiency can cause death by affecting the nervous system, the cardiovascular system, and the gastrointestinal tract^21^. In Lao PDR, it has been an important public health problem since the 1960s^22^; however, an increase in cases diagnosed with thiamine deficiency was found in both of urban and rural areas after the 1990s^19,20^. Thiamine deficiency can develop within a few months of a diet containing very poor sources of thiamine, such as polished rice^21^. In Lao PDR, most postpartum women traditionally have food restrictions^18,20^ and the percentage of 6–9-month-old infants who have weaning food other than rice is very low^23^. Breastfeeding until 24-months-old is recommended; but the thiamine content in breastmilk is associated with the mothers’ thiamine status^21^. Thiamine deficiency in breastfeeding mothers and pregnant women has also been reported^18^. Further, mortality attributed to thiamine deficiency peaks during the first 3 months of life^20^, and may contribute to the difference between IMR and NMR. To prevent thiamine deficiency, nutrition education to women who visit health facilities for ANC and immunization for their babies should be provided.

In all women, auxiliary nurses as birth attendants compared to doctors, male infants and small size at birth compared to average were significantly associated with high infant mortality. Males have a biologically higher risk of mortality and morbidity than females, not only in the neonatal and infant periods but also throughout life^24–26^. The incidences of low birth weight and premature birth are also higher for male than female babies without the influence of maternal factors^26,27^. Auxiliary nurses are medical professionals established in 1975 to address the nursing shortage after the Laotian Civil War. The education for auxiliary nurses was conducted over 3–12 months at provincial training centers, while full nurses undertake at least 2 years of education in nursing schools or universities. To reduce maternal mortality, the government started a 5-week training program on birth assistance for auxiliary nurses in 2008, which became a 3-month program in 2013. The education of auxiliary nurses was terminated in 2015. However, 2275 auxiliary nurses and primary-level midwives still worked in public health facilities across the country in 2018, while the numbers of high- and mid-level nurses were 755 and 3910, respectively. To reduce infant mortality, currently employed auxiliary nurses should be upgraded to at least mid-level nurses through additional education or training.

When the data of women whose infants were average or large size at birth were analyzed, five factors were associated with high infant mortality in all three models. Two factors (auxiliary nurses as birth attendants and male infants) were the same in the analysis of all women. The three factors were pregnant at the time of the interview, 1–3 ANC visits compared to four visits or more, and having a postnatal baby check. A short interpregnancy interval increases the risk for preterm births; small for gestational age; and adverse fetal and infant outcomes, such as birth defects, neonatal mortality, and infant mortality^28–30^. Furthermore, in developing countries, a short interpregnancy interval can also increase the risk of maternal morbidity from third trimester bleeding, puerperal endometritis, and anemia^31^. These results suggest that short interpregnancy intervals could be causing higher IMRs in Lao PDR by negatively affecting maternal health conditions. One explanation for this is that, with a short interpregnancy interval, maternal nutritional status does not fully recover from the demands of the previous pregnancy, delivery, and breastfeeding^32,33^. These results suggest that increasing availability of contraception would be an important intervention to increase the interpregnancy interval.

The current results suggest that four ANC visits or more are needed to improve the IMR. The Lao government set the target of the coverage of at least one visit and four visits of ANC as 90% and 75%, respectively, by 2020^34^. The ANC coverage improved from 37.5% in 2006^35^ to 54.2% in 2012^36^ and 79.1% in 2017 (this study). The coverage of four ANC visits also increased from 36.9% 2012^36^ to 61.8% in 2017 (this study). Many kinds of service should be provided in ANC: check of maternal health condition and fetal well-being, human immunodeficiency virus (HIV) test, tetanus injection, anemia test, provision of iron supplement or malaria drugs, as well as education on MCH. Therefore, 1–3 visits of ANC are insufficient to support MCH. There is a need to increase the quality of ANC and maternity care services more broadly^34^.

In this study, the percentage of women who had postnatal baby checks was only 13.5%, which was much lower than the ANC coverage or the immunization coverage in children^37^. The major diseases in infants are infectious diseases, particularly pneumonia and diarrhea^38,39^. Having to travel long distances to hospitals, road conditions, and lack of transportation are barriers to accessing health services in Lao PDR^40^. These factors suggest that Lao women may only take their babies to the checks when they are moderately or severely ill.

This study used more variables compared to previous studies including women’s attitudes toward domestic violence. Obstetrical and neonatal factors, but not socio-demographic factors, were associated with infant mortality. A previous study in Lao PDR showed that maternal age, living in a rural area, and poor economic status were independent risk factors for high IMR^35^. This means that government strategies to improve poverty may be effective for improving IMR. However, the current results indicate that the government should strengthen and improve the quality of MCH service, especially its contents and providers.

This study had some limitations. First, recall bias might have occurred, as the women reported on their behaviors and experiences in the previous 2 years. Second, babies’ size at birth was used rather than birth weight, as 34.2% of women did not know or respond to the question about birth weight. This may be because 96.0% of the women gave birth at locations other than health facilities. Moreover, the percentages of babies with small (8.9%), average (75.6%), and large (13.7%) birth sizes were very similar to the birth weight categories of < 2500 g (11.0%), 2500–3500 g (77.7%), and > 3500 g (11.3%), respectively, among babies who were weighed at birth in this study. Third, women whose data were missing were excluded; however, these women might have experienced greater infant mortality.

## Conclusions

In Lao PDR, the estimated IMR decreased from 191 per 1000 live births in 1978–1987 to 39 in 2017, and the difference between the IMR and NMR did not change after 2009. Among all women, factors associated with high infant mortality were auxiliary nurses as birth attendants compared to doctors, male babies, and small-sized babies at birth. Among women whose babies were average or large at birth, auxiliary nurses as birth attendants, male babies, pregnancy at the time of the interview, 1–3 ANC visits compared to four visits, and having postnatal baby checks were factors associated with high infant mortality. These results suggest that the Ministry of Health should strengthen MCH by improving the content and quality of ANC and upgrading auxiliary nurses to at least mid-level nurses by providing additional training and education. Family planning and health education for all citizens also need to be strengthened.

## Methods

### LSIS-II

This was a cross-sectional study using the secondary data of women and their live births from the LSIS-II. The LSIS-II was a nation-wide survey, including 22,287 households across all 18 provinces in Lao PDR in 2017, which was conducted by the National Lao Statistics Bureau, the Ministry of Planning and Investment, the Ministry of Health, and the Ministry of Education and Sports—with technical support from the global and regional Multiple Indicator Cluster Survey team from the United Nations Children’s Fund and other international agencies^8^. The survey steering committee developed an ethical protocol to ensure participants’ rights. The study protocol was approved by the Ethics Committee of the Ministry of Planning and Investment in May 2016.

The total number of households in 2017 was 1,244,010^9^. A multi-stage, stratified cluster was employed for sampling to ensure that the survey population represented a reliable estimation of indicators in urban and rural areas throughout the country. Urban and rural areas within 18 provinces were decided as the sampling strata, and the sample selection was performed as reported^8^. Weight calculations were used to ensure data accuracy, considering non-response cases for bias prevention. Of the 22,287 households included in the survey, 25,305 women aged 15–49 participated in interviews from July to November 2017. Verbal informed consent was obtained from all women. Trained interviewers interviewed all women separately to complete individual questionnaires^41^, which consisted of women’s demographics, mass media, fertility/birth history, desire for last birth, maternal and newborn health, postnatal health checks, contraception, unmet needs, attitudes toward domestic violence, marriage, sexual behavior, HIV, tobacco and alcohol use, and anemia testing^8^. An Hb test was conducted for women in 50% of the sample households (when women consented). A drop of blood from a finger prick was drawn into a microcuvette, and Hb analysis was conducted on-site with a battery-operated portable analyzer.

### Estimation of IMR and NMR

All 25,305 women responded to questions about their live births, which were 54,163 children who were born from 1978 to 2017: birth dates; whether the children were alive or dead at the time of the interview; and, if a child was dead, the age at the time of death. Among 54,163 children, 436 children had missing data, and the data from 53,727 were analyzed. The IMR and NMR for each year were estimated using the said data, such as the total number of live births, neonatal deaths, and infant deaths. The total number of live births from the women each year increased from 1978 to 1999, and it was approximately 2000 in 2000–2016 and 1498 in 2017. The IMR and NMR from 1978 to 1987 were estimated as the average in the period but not each year because the total live births in each year was small.

### Identification of factors associated with infant mortality

In the LSIS-II, only women who had live births in the 2 years before the interview responded to questions about their usage of healthcare services during their last pregnancies and for their last children. Of all 25,305 women, 4460 women had live births in the 2 years before the interview. To study factors associated with infant mortality, we included 2189 women with live births in the 2 years before the LSIS-II interview whose last child was a singleton, and whose last living child was aged at least one at the time of the interview or whose last child had died before the interview. Women who did not provide the last live birth date or whose infant was alive but aged less than one were excluded.

### Variables and measurements

We used socio-demographic data including age, region, residence, ethno-linguistic group, education level, wealth index quantile, marital status, husbands’ age, and age at first marriage. Obstetrical data included information on the following: parity; history of miscarriage, stillbirth, and abortion; current pregnancy status; history of smoking and drinking; Hb level at the time of the interview; desire to have the last baby; number of ANC visits; ANC provider; iron supplementation and tetanus toxoid vaccination during the last pregnancy; delivery location; birth attendant; and mode of delivery. Infant data included information on sex, infant size at birth, drink given to babies in the first 3 days after birth, history of baby checks, and age at death if the infant died before turning one. Residence comprised three categories (urban, rural with roads, and rural without roads) concerning the villages where women lived, which were defined by the criteria of urban villages and rural villages^42^. The ethno-linguistic group that women belonged to were categorized into Lao-Tai, Hmong-Mien, Mon-Khmer, Chinese-Tibetan, and other^43^. Questions concerning women’s history of smoking and drinking were “have you ever tried cigarette smoking, even one or two puffs?” and “have you ever drunk alcohol?”^8^ Infant size at birth was based on the mothers’ perceptions, because only 65.8% of women provided the birth weight of their infants. Five responses were categorized into three such as average, small (very small and smaller than average), and large (very large and larger than average). Domestic violence during pregnancy and women’s attitudes toward it are associated with maternal outcome and child mortality^44,45^. Data concerning attitudes toward domestic violence included women’s perceptions of husbands’ violence toward their wives in the following scenarios: if a wife goes out without telling her husband, if a wife neglects her children, if a wife argues with her husband, if a wife refuses sex with her husband, or if a wife burns food.

### Statistical analysis

Data analyses were performed using SPSS version 25.0 (IBM SPSS Inc.; New York, USA). Sampling weights were applied in all analyses except for survival time to ensure the natural representation of the sample for all strata. A Kaplan–Meier curve was constructed to estimate the survival time. A logistic regression model was used to obtain an odds ratio with a 95% confidence interval. In multivariate analysis, three methods were applied. In the forced-entry method (Model 1), all variables were included. In Models 2 and 3, variables were selected in the forward and backward stepwise selection, respectively. The criteria for variable selection were P < 0.05 for adding variables, and P > 0.1 for removing variables. P-values < 0.05 were considered significant. In this study, written informed consent was waived due to a secondary analysis of the anonymous data of the LSIS-II (in accordance with the study protocol of the LSIS-II approved by the Ethics Committee of the Ministry of Planning and Investment). All methods were performed in accordance with the relevant guidelines and regulations.
